# Tungsten as a Chemically-Stable Electrode Material on Ga-Containing Piezoelectric Substrates Langasite and Catangasite for High-Temperature SAW Devices

**DOI:** 10.3390/ma9020101

**Published:** 2016-02-06

**Authors:** Gayatri K. Rane, Marietta Seifert, Siegfried Menzel, Thomas Gemming, Jürgen Eckert

**Affiliations:** 1 SAWLab Saxony, IFW Dresden, 270116 01171 Dresden, Germany; marietta.seifert@ifw-dresden.de (M.S.); s.menzel@ifw-dresden.de (S.M.); t.gemming@ifw-dresden.de (T.G.); 2 Erich Schmid Institute of Materials Science, Austrian Academy of Sciences (ÖAW), Austria; 3 Department Materials Physics, Montanuniversität Leoben, 12 A-8700 Leoben, Austria; juergen.eckert@unileoben.ac.at

**Keywords:** high temperature sensor, surface acoustic wave devices, electrode material, refractory metal thin film

## Abstract

Thin films of tungsten on piezoelectric substrates La_3_Ga_5_SiO_14_ (LGS) and Ca_3_TaGa_3_Si_2_O_14_ (CTGS) have been investigated as a potential new electrode material for interdigital transducers for surface acoustic wave-based sensor devices operating at high temperatures up to 800 °C under vacuum conditions. Although LGS is considered to be suitable for high-temperature applications, it undergoes chemical and structural transformation upon vacuum annealing due to diffusion of gallium and oxygen. This can alter the device properties depending on the electrode nature, the annealing temperature, and the duration of the application. Our studies present evidence for the chemical stability of W on these substrates against the diffusion of Ga/O from the substrate into the film, even upon annealing up to 800 °C under vacuum conditions using Auger electron spectroscopy and energy-dispersive X-ray spectroscopy, along with local studies using transmission electron microscopy. Additionally, the use of CTGS as a more stable substrate for such applications is indicated.

## 1. Introduction

Surface acoustic wave (SAW) devices have exhibited promising application as sensors for monitoring and controlling high temperatures above 400 °C and are increasingly demanded due to their functionality provided by their small size, robustness, and unique capability for wireless interrogation under extreme environments [1,2,3,4]. However, for high-temperature applications above 500 °C, several issues are related to the choice of durable materials for the interdigital transducer (IDT) thin film electrode and the piezoelectric substrate for enabling long-term applicability, such as:

- Chemical and structural stability of film-substrate composite in harsh environments;
- Resistance to stress-induced damaging effects in the nanocrystalline thin film electrode, such as agglomeration, delamination, creep, *etc.* These effects are enhanced at higher temperatures due to the dissimilar coefficient of thermal expansion (CTE) of the materials in contact.
- Low and stable electrical resistivity of the electrodes at the operating temperatures.

For over a decade, the piezoelectric substrate La_3_Ga_5_SiO_14_ (LGS) has been investigated extensively at high temperatures for application in SAW sensors in harsh environments under an air atmosphere [5,6,7,8,9,10]. However, recent studies have revealed chemical instability of LGS and its high sensitivity to different surrounding atmospheres. Especially under vacuum or at low oxygen partial pressures, a high rate of diffusion of oxygen and gallium atoms was observed already at about 700 °C [11,12,13,14]. This diffusion also affects the metal electrode, e.g., oxidation of Ir IDT upon annealing in vacuum was observed due to the diffusion of oxygen from the LGS substrate into the Ir thin film [13]. In addition to being unstable in vacuum, recent studies on Pt-based IDTs on LGS exhibit the presence of gallium in the electrode film also upon annealing in air [15]. Deposition of a covering alumina layer on top of the LGS substrate hindered the diffusion of Ga/O out from the substrate to a certain extent in this case. Ga and Pt can form various phases according to the phase diagram [16], suggesting that the inherent instability of the LGS substrate could be one of the reasons for the deterioration of the Pt films.

It is well-established now that diffusion of Ga and O takes place during high temperature annealing in low-oxygen partial-pressure environments. In addition, studies by Aubert *et al.* [17] have also shown that severe surface modification which revealed itself as high density of visible flaws on the surface were observed upon annealing bare LGS substrates in air at 1200 °C. These flaws were severely Ga-depleted. Bardong *et al.* [11] observed the degradation of the crystals when annealed in both air and vacuum. Degradation in vacuum occurred already at 480 °C and was attributed to the loss of gallium oxide. Recent studies in our group present a detailed investigation on LGS substrate upon annealing at 800 °C under vacuum [18]. Substrate modification by the formation of microcracks on the surface with strongly altered chemical composition was evidenced.

Another high-temperature stable piezoelectric substrate of the same LGS family is Ca_3_TaGa_3_Si_2_O_14_ (CTGS) [19,20,21]. This material has better piezoelectric properties, such as higher effective piezoelectric coefficient at high temperature. The crystals have better quality due to the lower Ga content, higher electrical resistivity, higher mechanical strength and a lower thermal expansion anisotropy as compared to langasite [20,21,22,23]. This relatively newer substrate material is lesser known and thin films on it are not extensively studied yet. Our studies on RuAl films [24] and W films [25] on CTGS showed that while the latter system was stable up to 800 °C, the former was degraded similar to that on LGS, but to a lesser extent.

In this paper, the in-depth studies on tungsten thin films as IDT material on piezoelectric substrates of LGS and CTGS is presented up to 800 °C under vacuum. The choice of refractory metal W, over the commonly used inert Pt, is based on it having a high melting point (3422 °C) which would lead to minimal creep-related damaging effects (the activation energy for self-diffusion at 1000 °C for W is ~ 6 eV while for Pt is ~ 3 eV [26]), its lower electrical resistivity (5.49 μΩ ·cm) as compared to Pt (10.6 μΩ·cm), closer CTE (4.5 × 10^−6^ K) to LGS (a_11_ = 5.63 × 10^−6^ K) and CTGS (a_11_ = 3.3 × 10^−6^ K) [20] than for Pt (8.8 × 10^−6^ K). In addition, W has exceptional thermal shock resistance due to its low CTE combined with its high thermal conductivity (174 W·m^−1^·K^−1^) which for Pt is much lower (72 W·m^−1^·K^−1^) [27].

Former studies in the group have been performed on W, W/Mo, and RuAl systems as electrode material on LGS and CTGS [25,28]. Upon vacuum annealing of the films up to 800 °C, oxidation of the RuAl and presence of Ga in the film was detected for the films on both LGS and CTGS. In contrast, the extent of degradation of W on LGS was much lower, while a very high stability was observed for W on CTGS. The degradation of RuAl film was attributed to the diffusion of Ga/O from both the substrates into the thin film upon vacuum annealing. Since W was unaffected by Ga/O diffusion, we delved deeper to gather an understanding of the properties of the film-substrate upon annealing in vacuum by detailed investigations using Auger electron spectroscopy and transmission electron microscopy. The results of the local analysis of the films are provided in this short communication.

The application of the electrodes is presently restricted to vacuum and inert atmospheres since application in different environments would require a passivating layer to avoid degradation of the electrode. Thus, low-cost materials exhibiting high-temperature stability, such as W or Mo, are studied rather than the Pt-based electrodes. Our studies are directed towards lab-scale applications in vacuum process chambers (thin film deposition chambers where SAW sensors can be attached to the substrate) or where precise and real-time temperature measurements are required (phase transformation studies, reaction thermodynamics, *etc.*). In addition to these, applications can also be thought in other fields which perform tests in vacuum, e.g., NASA uses thermo-vacuum chambers for conducting tests on aircraft and spacecraft systems and components [29].

## 2. Experimental Section

100 nm films of W were deposited by DC magnetron sputtering onto LGS and CTGS substrates at 400 °C under high vacuum conditions (at a pressure of 1.8 × 10^−4^ Pa from a 99.95% pure target using 99.999% Ar sputtering gas). The commercially available (FOMOS-Materials, Moscow) LGS (138.5° Y-cut wafer of 4” diameter) and CTGS (90° Y-cut wafer of 3” diameter) wafers of 500 µm thickness (surface finish according to IEC 2005) were used as obtained. The films were deposited onto 10 × 10 mm cut wafer pieces thereof. The samples were annealed at 400, 600, and 800 °C each for 12 h under high vacuum (10^−3^ Pa) conditions. All the measurements have been performed at room temperature after the annealing process.

The films have been investigated by X-ray diffraction (XRD, Philips (now PANalytical) X’Pert PW3040/00, Co-Kα, PANalytical, Almelo, The Netherlands) in Bragg-Brentano geometry. Depth profiles of the atomic concentration were recorded using Auger electron spectroscopy (AES, JEOL JAMP-9500F Field Emission Auger Microprobe, 1 kV Ar^+^ ions at 0.7 µA, JEOL, Tokyo, Japan).

The surface of the substrates and substrate-film systems were analyzed by scanning electron microscopy (SEM, Zeiss Ultra Plus, Carl Zeiss Microscopy GmbH, Jena, Germany) and atomic force microscopy [AFM, DI Dimension 3100, Bruker (formerly Digital Instruments), Billerica, MS, USA]. Cross sections of the samples have been prepared by the focued ion beam technique (FIB, Zeiss 1540 XB CrossBeam, Carl Zeiss Microscopy GmbH, Jena, Germany).

High-angle annular dark-field scanning transmission electron microscopy (HAADF-STEM, FEI Technai F30, FEI, Hillsboro, OR, USA) was performed to analyze the microstructure on the nanoscale. Together with energy dispersive X-ray spectroscopy (EDX, EDAX, Mahwah, NJ, USA) in the same instrument, the local chemical composition has been determined.

## 3. Results and Discussion

**Figure 1:**
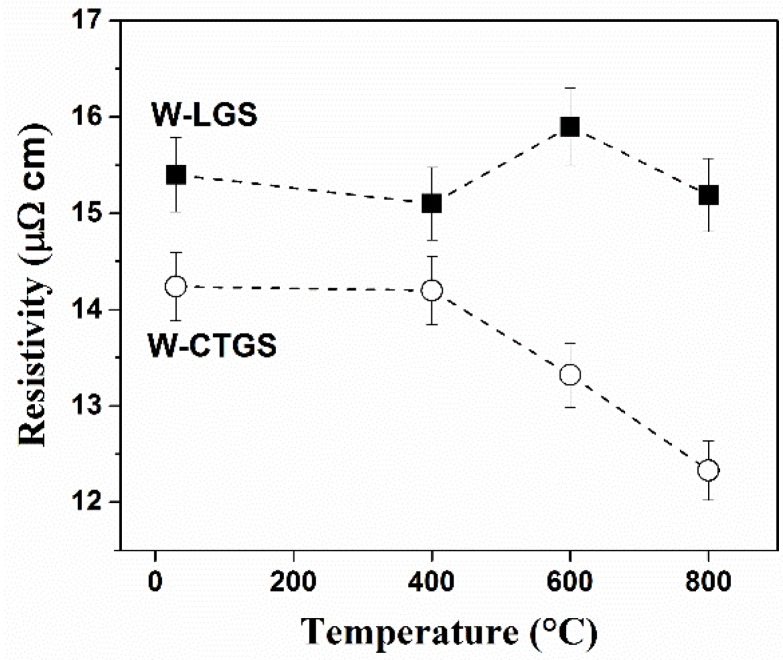
Electrical resistivity upon annealing 100 nm·W films on LGS and CTGS at 400 °C, 600 °C, and 800 °C for 12 h each under vacuum (a maximum error of < 5 % is considered due to fluctuation in the voltage).

X-ray diffraction measurements show that the as-deposited W films on both the substrates consist of predominantly {110} oriented α-W grains with the out-of-plane coherently diffracting domain size of about 35 nm [30]. The electrical resistivity (van der Pauw method) of the as-deposited W film on LGS was 15.4 µΩ·cm and that on CTGS was 14.3 µΩ·cm. The electrical resistivity of the W films after vacuum annealing at 400, 600, and 800 °C for 12 h each are shown in Figure 1 (measurements were performed at room temperature after the particular annealing steps).

The samples annealed at 400 °C exhibit no change in the resistivity. Upon annealing to 600 °C, while the resistivity of W on CTGS reduced, no change was observed for the films on LGS. The final resistivity of W-LGS after annealing to 800 °C was almost unchanged, whereas approximately 10 % reduction was observed for W-CTGS.

XRD measurements after annealing W-LGS at 600 °C and 800 °C for 12 h, show a continual sharpening of the (100) reflection. Single line analysis [31] on this sample indicated an increase of the grain size. Alongside, the intensity of other reflections increases indicating the loss of any preferred orientation. Similar to W-LGS, the reflections of the W film on CTGS also become narrower (due to grain growth and defect annihilation); however, a sharper {110} preferred orientation of the grains develops in this sample. Grain growth and improved grain orientation should lead to the decrease of the electrical resistivity in the W films on CTGS.

**Figure 2:**
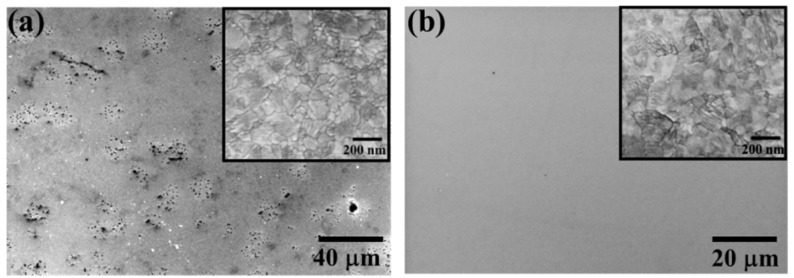
Low magnification SEM images of W films on (**a**) LGS and (**b**) CTGS post-annealing at 800 °C for 12 h under vacuum (high-magnification images are shown in the inset).

In order to understand the indiscernible electrical resistivity evolution of W on LGS upon annealing, microstructural investigations were carried out on the samples heated up to 800 °C using SEM. Low magnification SEM images of the W-LGS system (Figure 2a) showed severe surface damage manifested as scattered blisters over the entire film surface. In contrast, no defects were observed on the W-CTGS film surface (Figure 2b) and the surface roughness (RMS value) as measured by atomic force microscope (AFM, DI Dimension 3100) was 1.7 nm for this film. The microstructures of the two films, however, are similar in the non-defected regions (see inset in Figure 2 for high-magnification SEM images).

**Figure 3:**
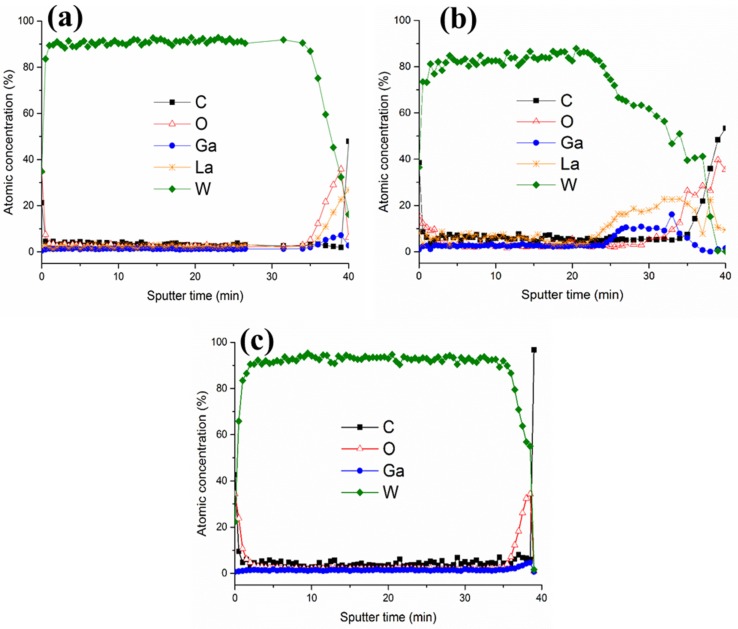
AES depth profiles made at (**a**) non-defected region on W-LGS, (**b**) around the blister location on W-LGS, and (**c**) on W-CTGS.

AES measurements were performed on these two samples (Figure 3) to gather information on the presence of contaminants in the films. Samples were rotated to minimize roughening effects. Measurements on W-LGS were performed at different locations, *i.e.*, at the blistered location as well as on the smoother region. Measurements at the blistered locations (shown in Figure 3b), shows the presence of La and Ga in addition to C and O in the depths of the film. Depth profiling the blistered location showed a high carbon content (~5 at.%) within the film. However, measurements at the smoother regions (shown in Figure 3a) show surface contamination of only C and O and no contamination in the depths of the film. In comparison, the depth profile of the W-CTGS sample at several locations show no contaminants within the film (Figure 3c). The surface of this film shows the presence of only C and O. The presence of La and Ga near the surface regions at the blistered locations on W-LGS indicates that the substrate undergoes changes.

**Figure 4:**
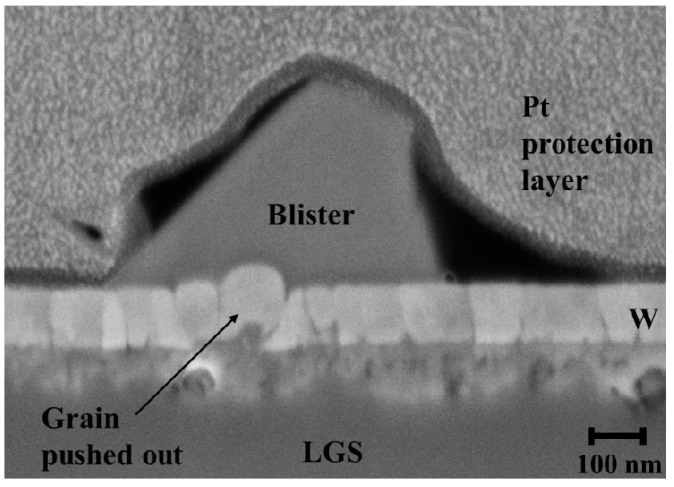
FIB-cut around the blistered area on W-LGS after annealing to 800 °C showing a grain pushed out.

Thus, several FIB-cuts were made at the blistered region, perpendicular to the film surface, on the annealed W-LGS sample to understand the origin of these blistered defects. Figure 4 shows one such FIB-cut with a visible protruding “blister”. It can be seen from this figure that the substrate region just below the film has varying contrasts indicating a change in the substrate composition. Furthermore, a grain (see image) is seen pushed upwards close to this blister region. Sequential FIB-cuts in this region showed that the blister originated from the substrate leading to not only “pushing-out” of individual grains but also disrupting regions in the deposited film and the loss of continuity of the film on the substrate. Thus, the W film was absent at the regions of the blister due to the outwards flow of the substrate material.

**Figure 5:**
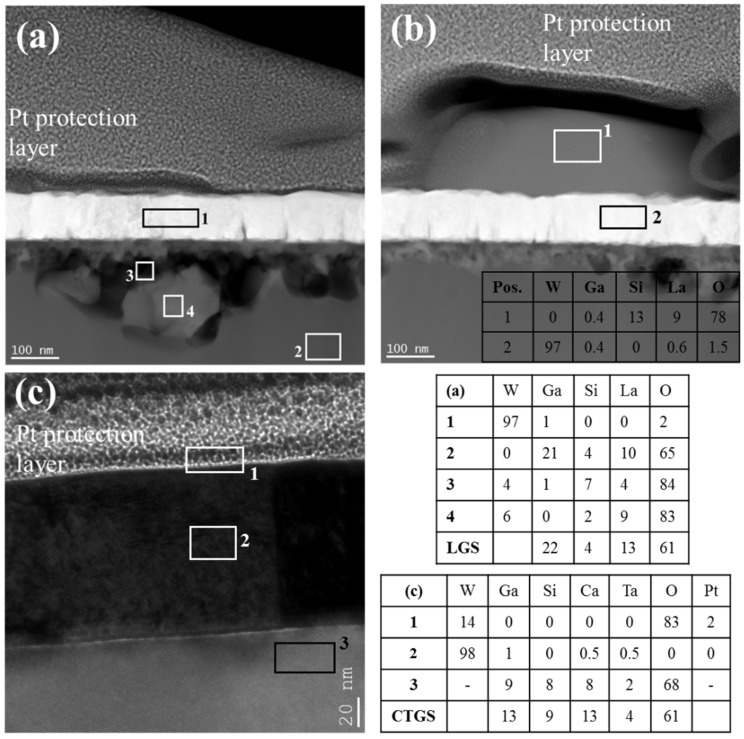
TEM images along with the composition (table inset) of the marked regions obtained by EDX analysis of (**a**) W-LGS; (**b**) W-LGS showing the blister; and (**c**) W-CTGS.

In order to understand these defects that developed on the film on LGS after annealing up to 800 °C, TEM studies were performed on a lamellae at the blistered surface region, as shown in Figure 5a,b. It can been seen in Figure 5a that while the film in this region looks intact, high contrasts are visible in the LGS substrate just below the film interface. A local analysis of the chemical composition with EDX on the substrate close to the film interface revealed severe changes in the composition of the substrate (see position 3 and 4 in Figure 5a, and the corresponding composition in Table (a)). In particular, these regions were either entirely depleted of Ga or had diminished Ga content. The stoichiometric composition of the substrate was retained in the depths of the substrate (position 2). Interestingly, the W film composition (Table (a), Region 1) is unaffected as was also suggested by the AES measurements. Further on, no oxidation of the W-LGS film can be detected from the EDX analysis. Another position on the same lamella is showed in Figure 5b having the blister as seen protruding on the film surface (Figure 5b, Region 1). This grey region on top of the film surface is composed of predominantly La, Si and O (inset table). As mentioned above, AES measurements at the blistered regions also showed the presence of La.

The TEM image of the W-CTGS system is shown in Figure 5c. Columnar grains of W spanning the film thickness and a sharp interface in between the film and the substrate was confirmed from a view of a number of images. EDX analysis (Table (c)) reconfirms the purity of the W film. The composition of the substrate obtained from EDX conforms closely to that of stoichiometric CTGS single crystals. Thus, the W-CTGS system is rather stable with respect to the film structure, as well as the chemical stability of the substrate.

The destroyed architecture of the W-LGS system can be understood to be a consequence of the instability of LGS substrate under vacuum conditions leading to severe surface modifications, as was already reported by us [18]. Upon annealing, several microcracks on the substrate were visible (Figure 4 in reference [18]). When material with high affinity to oxygen was deposited on LGS, such as RuAl, and annealed in vacuum, severe oxidation of the film took place, and the presence of Ga in the film was detected. In contrast to RuAl, in the present study, although visual damages in the film and substrate can be identified, the structural damages are independent of chemical changes in the W film (as confirmed by EDX and AES). This could be attributed to the relatively low affinity of oxygen to W as compared to for Ga. One possible explanation for this could be the higher stability of the gallium oxide [32] as compared to tungsten oxide [33] at low oxygen partial pressures, according to the Ellingham diagram. Additionally, W and Ga (as well as La) are mutually immiscible and do not form any phases, according to the phase diagram [34].

Thus, upon annealing W films on LGS, under vacuum, blister-like features were observed which originate at the substrate as a result of gallium oxide diffusion. The material displaced from LGS goes through the film at the grain boundaries (easy diffusion paths) disturbing the film continuity. Such local defects in the film offer additional scattering contribution to the flow of electrons and, hence, can explain the anomalous resistivity evolution on LGS.

In comparison to LGS, we observe that the CTGS substrate (which has lower Ga content and is a more ordered structure) is more stable for applications under vacuum conditions. The W film-CTGS substrate composite are stable, chemically and structurally, up to 800 °C under vacuum.

## 4. Conclusions

W films on LGS develop defects when annealed under vacuum due to the instability of the LGS substrate with respect to gallium oxide diffusion. The chemical changes in LGS were accompanied by the development of structural defects in the substrate that led to the deterioration of the W film architecture on LGS. The chemical instability of the LGS substrate was, however, inconsequential to the chemical stability of the W film. However, the inherent instability of the LGS substrate would pose a problem in its utilization for high-temperature applications. In contrast to LGS, the CTGS substrate with the W film on it were stable, chemically, as well, as structurally, up to a temperature of 800 °C under vacuum. Thus, tungsten, with its relevant properties as an electrode material, is an interesting candidate for IDTs on the Ga-containing substrate LGS and CTGS for application under vacuum up to 800 °C.
